# Supplementary material for: The E3 ubiquitin ligase IDOL regulates synaptic ApoER2 levels and is important for plasticity and learning
Source: eLife. 2017 Sep 11;6:e29178. doi: 10.7554/eLife.29178 (PMC5593505; doi:10.7554/eLife.29178)
Supplement: Transparent reporting form [file elife-29178-transrepform.docx]

***eLife’s* transparent reporting form**

We encourage authors to provide detailed information *within their submission* to facilitate the interpretation and replication of experiments. If you have any questions, please contact us: [editorial@elifesciences.org](mailto:editorial@elifesciences.org).

**Sample-size estimation**

- You should state whether an appropriate sample size was computed when the study was being designed
- You should state the statistical method of sample size computation and any required assumptions
- If no explicit power analysis was used, you should describe how you decided what sample (replicate) size (number) to use

Please outline where this information can be found within the submission (e.g., page numbers or figure legends), or explain why this information doesn’t apply to your submission:

For primary neuron culture experiment, we conducted the experiments 2-3 times (biological replication) from different batch of neuron culture. Within individual experiment, each experimental condition is represented by the samples or pooled samples from duplicate or triplicate wells. The sample size information can be found in the Figure legends;

For protein sample analysis, the representative samples are individual sample or pooled samples from 4-8 mice. The detailed sample size information can be found in the figure legends;

For mouse experiment related to behavior test, repeated measures ANOVA or one way ANOVA were used for the statistical comparisons between genotypes. P value smaller than 0.05 is considered significant. Based on our pilot experiments, the standard deviation is estimated within 20% of the mean value. In order to detect a significant (P<0.05) difference with a power of 90%, we need at lease 9 mice per group. The detailed sample size information can be found in figure 8 legend

**Replicates**

- You should report how often each experiment was performed
- You should include a definition of biological versus technical replication
- The data obtained should be provided and sufficient information should be provided to indicate the number of independent biological and/or technical replicates
- If you encountered any outliers, you should describe how these were handled
- Criteria for exclusion/inclusion of data should be clearly stated
- High-throughput sequence data should be uploaded before submission, with a private link for reviewers provided (these are available from both GEO and ArrayExpress)

For primary neuron culture experiment, we conducted the experiment 2-3 times (biological replication) from different batch of neuron culture. Within individual experiment, each experimental condition is represented by the samples or pooled samples from duplicate or triplicate wells (technique replications). The detailed information has been provided in the figure legends.

This study did not include high-throughput sequencing data.

Please outline where this information can be found within the submission (e.g., page numbers or figure legends), or explain why this information doesn’t apply to your

**Statistical reporting**

- Statistical analysis methods should be described and justified
- Raw data should be presented in figures whenever informative to do so (typically when N per group is less than 10)
- For each experiment, you should identify the statistical tests used, exact values of N, definitions of center, methods of multiple test correction, and dispersion and precision measures (e.g., mean, median, SD, SEM, confidence intervals; and, for the major substantive results, a measure of effect size (e.g., Pearson's r, Cohen's d)
- Report exact p-values wherever possible alongside the summary statistics and 95% confidence intervals. These should be reported for all key questions and not only when the p-value is less than 0.05.

For in vitro electrophysiology, The Mann–Whitney U test was chosen for statistical comparisons of the LTP/LTD data using Origin Pro 9.0 software (OriginLab Corporation, USA). A p value < 0.01 was considered significant.

For behavior tests, repeated measures ANOVA or one way ANOVA were used to analyze the data.

For Optical intrinsic signal imaging, A two-way ANOVA, followed by Bonferroni’s multiple comparison test was used to compute statistical differences between WT and *Idol^-/-^* groups in Origin Pro 9.0 software (OriginLab Corporation, USA). All data are presented as the mean ± standard error of the mean. Significance was set at p < 0.01. Raw data for Optical intrinsic signal imaging (data point from individual mouse) are provided in the figure 7

Detailed information is provided in the ‘Material and Methods’ section

Please outline where this information can be found within the submission (e.g., page numbers or figure legends), or explain why this information doesn’t apply to your submission:

(For large datasets, or papers with a very large number of statistical tests, you may upload a single table file with tests, Ns, etc., with reference to page numbers in the manuscript.)

**Additional data files (“source data”)**

- We encourage you to upload relevant additional data files, such as numerical data that are represented as a graph in a figure, or as a summary table
- Where provided, these should be in the most useful format, and they can be uploaded as “Source data” files linked to a main figure or table
- Include model definition files including the full list of parameters used
- Include code used for data analysis (e.g., R, MatLab)
- Avoid stating that data files are “available upon request”

Please indicate the figures or tables for which source data files have been provided:

None
